# Vancomycin Heteroresistance in Methicillin-resistant *Staphylococcus aureus*, Taiwan

**DOI:** 10.3201/eid1009.040239

**Published:** 2004-09

**Authors:** Jiun-Ling Wang, Sung-Pin Tseng, Po-Ren Hsueh, Keiichi Hiramatsu

**Affiliations:** * National Taiwan University Hospital, Taipei, Taiwan;; † Juntendo University School of Medicine, Tokyo, Japan

**Keywords:** Vancomycin, Heteroresistance, Staphylococcus aureus, Taiwan, letter

**To the Editor:** In 1997, Hiramatsu and colleagues reported the first clinical isolate of methicillin-resistant *Staphylococcus aureus* (MRSA) showing reduced susceptibility to vancomycin (*1*). Soon thereafter, vancomycin-intermediate *S. aureus* (VISA) or heteroresistant VISA was reported to have disseminated in Japanese hospitals (*2*). In Taiwan, a survey of >5,000 clinical isolates of *S. aureus* at one tertiary medical center from 1999 to 2001 showed negative results for VISA or vancomycin-resistant *S. aureus* (VRSA) (*3**,**4*). We report the first two isolations of heteroresistant VISA in Taiwan.

In June, 2000, an 89-year-old man (patient A) with a history of cerebrovascular accident underwent ileal resection for ischemic bowel disease, and primary MRSA bacteremia developed during the hospitalization. Vancomycin was given for 14 days, and his fever rapidly abated. In October 2000, a Port-A-Cath (Smiths Industries Medical Systems, Deltec, Inc., St. Paul, MN) was inserted in the left subclavian vein. On June 14, 2001, he had another blood isolate of MRSA during an episode of *Enterococcus faecalis* bacteremia. Fever resolved after 4 days of intravenous vancomycin treatment (1 g every 12 h), and vancomycin treatment was continued for 21 days. Subsequent culture of blood drawn from the Port-A-Cath and peripheral veins on June 29 and July 6, 2001, did not yield any organism. MRSA bacteremia relapsed in November 2001, and the patient received intravenous teicoplanin treatment (400 mg every 2 days) for 3 weeks, and fever resolved rapidly. Transthoracic echocardiograpic tests showed no vegetation on the cardiac valves. The patient was hospitalized again in March 2002 because of relapsing MRSA bacteremia. The Port-A-Cath was removed, and culture of fluid from the indwelling pocket yielded MRSA. Fever and MRSA bacteremia persisted, with 17 sets of positive blood culture from March to May 2002, even under an adequate dose of intravenous vancomycin (serum trough level of vancomycin = 9–24 µg/mL and serum peak level = 18–30 µg/mL) and rifampin (600 mg/day). An infected thrombus over the subclavian vein was detected by venous duplex and thought to be an unresolved focus. Linezolid (600 mg every 12 h) was given for 10 days (April 30–May 9, 2002) but discontinued because of progressive thrombocytopenia. Vancomycin and rifampin were resumed on May 10, and positive blood culture with MRSA was noted on May 14. Oliguric renal failure developed in the patient on May 21 followed by shock, and he died on May 23.

A 72-year-old man (patient B) with coronary artery disease and chronic renal insufficiency underwent coronary artery bypass grafting and aortic grafting for abdominal aortic aneurysm in December 1999. The postoperative course was complicated with second-degree atrioventricular block and progressive renal failure. He was implanted with a permanent pacemaker and started long-term hemodialysis in March 2000. In April 2000, catheter (double lumen for hemodialysis)-related MRSA bacteremia developed in the patient. Vancomycin (1 g/week) was administered and the catheter was changed, but 17 successive episodes of MRSA bacteremia recurred from May to July 2000, despite an adequate serum level of vancomycin (trough level = 13–21 µg/mL and peak level = 24–38 µg/mL). Transesophageal echocardiography showed vegetation on the tricuspid valve. Vancomycin was changed to teicoplanin (400 mg every 3 days) because of vancomycin-associated skin rashes and eosinophilia, and the treatment was continued for 1 month. No MRSA infection was found in the subsequent 6 months. In January 2001, a subcutaneous abscess and osteomyelitis developed over the right humerus after the patient was injured in a fall, and bacteremia subsequently developed. Local debridement was performed, and glycopeptides were given for 6 weeks (vancomycin for 12 days and teicoplanin for 30 days). From March to April 2001, he had repeated episodes of MRSA bacteremia associated with pus discharge from the pacemaker insertion site. The pacemaker was removed, and high-dose teicoplanin (600 mg every 3 days) was given for 4 months, during which time MRSA bacteremia did not recur.

MICs of vancomycin were determined for the 21 isolates of MRSA from the two patients (Table) by the broth microdilution and agar dilution method using brain-heart infusion (BHI) agar or broth and Mueller-Hinton agar (MHA) or broth (BBL Microbiology Systems, Cockeysville, MD), according to the recommendations by the National Committee for Clinical Laboratory Standards (*5*). Vancomycin MICs were also determined by the Etest (AB Biodisk, Solna, Sweden) by swabbing 0.5 McFarland Standard on a BHI agar plate, and the results were read after incubation for 24 h. Mu3 and Mu50 were used as control strains. MICs of the following antimicrobial agents were also determined by using the agar dilution method: oxacillin (MHA plus 2% NaCl) and teicoplanin, fusidic acid, and linezolid (MHA).

**Table:** Characteristics of 21 methicillin-resistant *Staphylococcus aureus* isolates recovered from two patients with recurrent bacteremia^a^

Designation of isolate	Date of isolation (mo/day/year)	Vancomycin MIC (µg/mL)					Teicoplanin MIC (µg/mL)
		Etest	Broth microdilution		Agar dilution		Agar dilution
		BHI	BHI	MHB	BHI	MHA	MHA
Patient A (89-y-old man)							
A1	6/30/2000	3	2	1	2	2	2
A2	6/14/2001	4	3	1	2	2	2
A3	11/19/2001	4	4	2	2	2	2
A4	3/5/2002	4	3	1	2	2	4
A5	3/22/2002	4	3	2	3	2	4
A6	4/3/2002	6	5	4	4	2	4
A7	4/14/2002	6	5	4	4	3	4
A8	4/29/2002	5	4	2	4	3	8
A9	4/29/2002	6	3	2	4	3	4
A10	5/7/2002	6	4	2	4	2	4
Patient B (72-y-old man)							
B1	4/26/2000	3	2	1	2	1	2
B2	5/6/2000	4	2	1	2	2	8
B3	5/18/2000	6	4	2	4	3	8
B4	5/29/2000	5	4	1	4	2	8
B5	6/15/2000	6	3	2	4	3	8
B6	6/24/2000	8	4	2	4	3	8
B7	7/19/2000	8	4	3	5	3	8
B8	1/9/2001	4	1	1	2	1	1
B9	1/26/2001	4	2	1	2	1	1
B10	3/21/2001	3	2	1	2	1	1
B11	4/6/2001	4	2	1	2	1	1
Mu3		3	3	2	3	2	-
Mu50		10	8	5	8	4	-

^a^BHI, brain heart infusion; MHB, Mueller-Hinton broth; MHA, Mueller-Hinton agar.

All 21 isolates were highly resistant to oxacillin (MICs > 128 µg/mL) but susceptible to linezolid (MICs = 1–2 µg/mL) and fusidic acid (MICs = 0.06–0.25 µg/mL). Isolates with reduced susceptibility to vancomycin (MICs > 4 µg/mL, determined by more than one method) included A6 and A7 from patient A and B7 from patient B. Two isolates (B6 and B7) had Etest vancomycin MICs of 8 µg/mL, and one of them also had an MIC of 5 µg/mL by the agar dilution method (Table).

Analysis of the vancomycin-resistant subpopulation of two MRSA isolates (A7 and B7) from the two patients, one isolate (isolate C, Etest vancomycin MIC = 4 µg/mL) of MRSA recovered from a patient with bacteremia in 2000, and Mu3 was performed according to the description by Hiramatsu et al. (1,2). Heteroresistant VISA refers to isolates with vancomycin MICs for one or more subpopulations above the susceptible range (i.e., > 4 µg/mL) (*1**,**2*). Isolates of A7 and B7, like the Mu3 strain, contained resistant subpopulations that grew in >4 µg/mL vancomycin and were thus considered as heteroresistant VISA strains (Figure).

**Figure:**
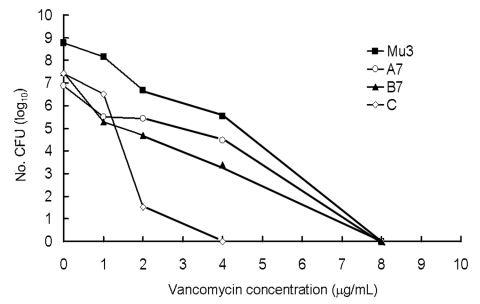
Population analysis of Mu3, two methicillin *Staphylococcus aureus* (MRSA) isolates (isolates A7 and B7) with heteroresistance to vancomycin, and one vancomycin-susceptible MRSA (isolate C).

Pulsed-field gel electrophoresis analysis after digestion of chromosomal DNA with *Xba*I showed that 10 isolates from patient A belonged to pulsotype a and those from patient B belonged to pulsotype b (Table). Heteroresistant VISA isolates were genetically indistinguishable from vancomycin-susceptible isolates.

This report is the first of heteroresistant VISA causing clinical infection in Taiwan, although the clinical importance of heteroresistant VRSA infection is unclear. While a previous report described no treatment failure of patients infected with heteroresistant VRSA strains (*6*), another study found higher death rate in patients infected with vancomycin-heteroresistant staphylococci (*7*). A recent case-control study of varying degrees of vancomycin susceptibility in MRSA bacteremia did not conclude whether a clinical difference was noted between bacteremia attributable to heteroresistant VISA and homogeneously susceptible strains (*8*). This report describes recurrent bacteremia caused by a single clone of MRSA that possessed subpopulations with different glycopeptide susceptibilities during different periods of treatment. These heteroresistant VISA strains were associated with prolonged glycopeptide use and glycopeptide treatment failure. Biofilm formation in the implanted intravascular devices may explain the relapsing nature in these two patients (*9*), and these heteroresistant VRSA strains might contribute to lack of bacteriologic eradication in infected valves and intravascular thrombi (*10*). Linezolid, having good in vitro activity against MRSA with reduced susceptibility to vancomycin, still failed to eradicate the organism within the infected thrombus in patient A.
